# Prevalence of high waist to hip ratio and its association with hypertension among married couples in India: A cross-sectional study

**DOI:** 10.1016/j.ajpc.2025.101302

**Published:** 2025-09-14

**Authors:** Rajeshwari A. Biradar, Jang Bahadur Prasad, Shiva S Halli

**Affiliations:** a School of Public Health, Sri Balaji Vidyapeeth (Deemed to be University), Puducherry, India; b Department of Biostatistics, Jawaharlal Institute of Postgraduate Medical Education & Research, Puducherry, India; c College of Community and Global Health, University of Manitoba, Winnipeg, Manitoba, Canada

**Keywords:** Waist-to-hip ratio, Indian couples, Hypertension

## Abstract

The study aims to explore the prevalence of high waist-to-hip ratio (WHR) and examine its association with hypertension and various socio-demographic factors among Indian couples with a focus on health implications. Data were extracted from the fifth round of the National Family Health Survey (NFHS) - a comprehensive population-based, cross-sectional survey conducted from 2019 to 2021. A total sample of 51,797 couples was analysed using bivariate and multivariable techniques to address the study objectives. The prevalence of high WHR among Indian couples was 36.3%. After adjusting for significant background factors, both female and male spouses had higher odds of hypertension when one spouse had a high WHR (female: OR = 1.19, p < 0.001, 95% CI: 1.13–1.25; male: OR = 1.30, p < 0.001, 95% CI: 1.22–1.38), compared to those with normal WHR. The risk increased when both spouses had high WHR, with odds of 1.44 in female spouses (OR = 1.44, p < 0.001, 95% CI: 1.37–1.52) and 1.56 in male spouses (OR = 1.56, p < 0.001, 95% CI: 1.47–1.66). The study highlights the significant health implications of high WHR in Indian couples. The findings emphasize the importance of monitoring WHR as an anthropometric measure for assessing hypertension risk and managing related health conditions in both clinical and public health settings.

## Highlights

• Data were extracted from the fifth round of the National Family Health population-based, cross-sectional survey.
• Around 36.3 % of Indian couples have high waist to hip ratio.
• High Waist to hip ratio in one spouse increases hypertension risk in both partners.
• Risk of hypertension is even higher when both spouses have high waist to hip ratio.
• Waist to-hip ratio should be used in routine screening to prevent hypertension.

## 1 Introduction

Being overweight is a well-established risk factor for a range of chronic conditions, including ischemic heart disease, diabetes, and certain types of cancer [1]. These conditions are major contributors to increased mortality, disability, and reduced quality of life [2,3]. Although spousal resemblance in weight status has been documented [4], there remains limited understanding of how familial and contextual factors such as shared dietary habits, physical activity patterns, and parenting styles as well as broader influences like socio-economic status, cultural norms, and access to healthcare shape the extent of this resemblance, particularly in relation to waist-to-hip ratio (WHR).

The differential impact of high WHR on metabolic complications within couples remains understudied. While studies from China have demonstrated a significant relationship between spousal BMI and the prevalence of being overweight [5], there is currently no comprehensive research examining WHR and its association with hypertension in married Indian couples.

Body Mass Index (BMI), though widely used, has notable limitations in assessing health risks associated with body fat distribution, as it does not differentiate between fat and muscle mass. Moreover, it fails to account for the distribution of body fat, particularly visceral fat, which is strongly linked to metabolic diseases. Research has shown that individuals with central (android) fat distribution face higher health risks compared to those with (gynoid) fat distribution [[6], [7]]. As a result, waist circumference (WC) and waist-to-hip ratio (WHR) have emerged as more accurate indicators of overweight and its associated health risks [[8], [9]].

Many studies have also found that the threshold values of WC vary by sex, age group, and ethnicity—for example, between men and women, adults and children, and Asian and non-Asian populations [[8], [9], [10],[3], [4],^8^]. In contrast, WHR incorporates a constant anthropometric dimension (height), allowing for adjustment of WC measurements. Moreover, the cut-off value of WHR has been shown to be relatively consistent across age, sex, and ethnicities [8,10].

Marital status has long been recognized as a significant social determinant of individual health outcomes and mortality [2,3]. Numerous studies have highlighted that married individuals are more likely to be overweight or obese, consume larger portions of food, and engage in less physical activity compared to their unmarried counterparts [[11], [12], [13]]. These differences may result from the environmental or lifestyle changes associated with marriage, which can exert both positive and negative effects on the health of couples [[14], [15]]. In India, high WHR and related disease conditions are becoming increasingly prevalent among couples, with a widening disparity in WHR measurements between partners [[2], [3]].

WHR is relevant in the context of metabolic disorders, as it is more strongly associated with various lifestyle factors and psychological health than BMI [16]. While there is substantial research on BMI and its association with health behaviours, the literature examining the combined influence of WHR and socio-economic factors, including lifestyle, in preventing hypertension in Indian married couples of the reproductive age group is limited [[17], [18]]. In this study, we aimed to understand the contribution of WHR to hypertension among Indian couples in the reproductive age group after controlling for socio-demographic factors.

## 2 Materials and methods

### 2.1 Sampling

Data from the fifth round of the National Family Health Survey (NFHS-5) conducted during 2019–21, was used in the study. NFHS-5 is a countrywide survey that covered 707 districts from 28 Indian States and 8 Union Territories. It provides representative information about maternal and child health indicators for all States and Union Territories of India (NFHS, 2019–21). We analyzed data on 51,797 couples for this study. The detailed methodology, with complete information on the survey design and data collection, has been published in the survey report [19].

### 2.2 Variables used in the study

For analysis, the primary outcome variable was hypertension with binary outcomes: hypertensive coded as 1, and normal coded as 0. Using the OMRON BP monitor, each respondent’s blood pressure was measured three times at 5-minute intervals by a trained health investigator. The last two measurements were averaged to determine the blood pressure of the individual. A person was classified as hypertensive if their average systolic blood pressure was higher than or equal to 140 mmHg, their average diastolic blood pressure was higher than or equal to 90 mmHg, or if they were currently taking prescribed medication to lower their elevated blood pressure. The hypertension status cut-off point was considered according to the National Family Health Survey Report [20]. The secondary outcome variable was WHR, however, it was also a primary explanatory variable for hypertension.

The independent variables were self-reported and these were socioeconomic and demographic factors such as age (15–24 years, 25–34 years, 35–44 years and 45–54 years), place of residence (urban or rural), highest educational level(categorized as no formal schooling, primary, secondary, and higher education), household wealth index variable based on household asset ownership, housing conditions, and amenities, categorized into poorest, poorer, middle, richer, and richest, religion(Hindu, Muslim and Others), caste(Scheduled Caste (SC), Scheduled Tribe (ST), Other Backward Class (OBC), and Others), occupation (not working, agricultural, skilled and unskilled manual and others) and Indian regions(North, Central, East, North East, West and South). The primary explanatory variable WHR was defined according to World Health Organization guidelines. We have simplified the explanation of the WHR formula and retained only the essential information needed to interpret the classification based on WHO thresholds. The WHR was defined by dividing the circumference of the waist by the circumference of the hips, using the formula:

WHR=WaistCircumference/HipCircumference

According to the World Health Organization (WHO), a healthy WHR varies by gender. For men, a WHR of 0.90 or less is considered healthy, while for women, a WHR of 0.85 or less is regarded as healthy [20]. This measurement is often used in health assessments, as it provides insight into an individual's risk of developing conditions related to excess abdominal fat, such as heart disease, type 2 diabetes, and other metabolic disorders. For surveys and studies, the recommended WHR measurement typically includes women aged 15–49 years and had not given birth in the two months prior to the survey, as well as men in the same age range [19,20]. Hence, the thresholds for unhealthy WHR have been specified as—WHR >0.90 for men and >0.85 for women was considered indicative of abdominal obesity. The other variable of interest was the couple's dietary habits. It is summarized by constructing a diet diversity index as follows:

### 2.3 Diet diversity index

While NFHS lacks detailed nutrient data, dietary diversity was inferred from food frequency. A Diet Diversity Index (DDI) was constructed using the frequency of consumption of healthy foods (e.g., pulses, green vegetables, fruits, milk, eggs, fish, meat) and unhealthy foods (e.g., fried items, aerated drinks) [21]. Frequencies were scored from 0 (never) to 3 (daily) for healthy foods, and reverse-scored for unhealthy items. The total score ranged from 0 to 27, categorized into “less diversified/unhealthy,” “moderately diversified,” and “diversified/healthy” diets. A diversified diet reflected frequent intake of healthy foods and limited unhealthy food consumption. The index was calculated separately for men and women, allowing simple cross-regional comparisons of diet diversity.

### 2.4 Statistical analysis

Data analysis was done using SPSS version 19.0. Bivariate analysis was performed to understand the prevalence of hypertension according to WHR and socio-demographic factors of the married couples. The risk of hypertension associated with WHR, and the background characteristics was inferred through logistic regression. The couples’ WHR and background characteristics were the major variables of interest to understand their association with hypertension. The results of the logistic regression were presented with a 95 % confidence interval (C.I).

## 3 Results

**Fig. 1:**
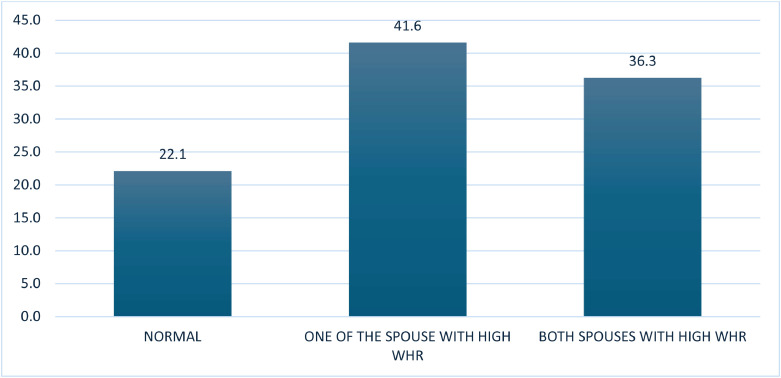
Prevalence of high waist-to-hip ratio among married couples in India, NFHS-5.

Out of 51,797 couples, 18,791 (36.3 %) had a prevalence of high waist-to-hip ratio (WHR). (Fig. 1). The overall prevalence of hypertension among female spouses was 43.7 % and among male spouses was 27.0 %, and the wide gap in the levels of hypertension between female and male spouses persisted across individual background characteristics. The prevalence of hypertension among both female and male spouses rose with age, with the highest rates in the 45–54 years age group for men (36.6 %) and 35+ years for women (53.3 %). An inverse association was observed between education and hypertension among female spouses. For instance, the female spouses with no formal schooling had the highest prevalence of hypertension (47.4 %) and the lowest prevalence among the highest educational category (39.0 %). But a reverse trend was observed between education and hypertension among the male spouses; however, the prevalence levels were lower, ranging between 25.5 % and 28.6 % compared to their female counterparts. (Table 1).

**Table 1:** Prevalence of hypertension in female and male spouses stratified by WHR and background characteristics among married couples in India, NFHS-5.

Variable		Hypertension in Female Spouses			Hypertension in male Spouses		
		No	Yes	Total	No	Yes	Total
Male Spouse age groups	15–24	75.0	25.0	2137	89.5	10.5	2137
	25–34	67.4	32.6	14,890	81.9	18.1	14,890
	35–44	54.6	45.4	19,275	72.1	27.9	19,275
	45–54	45.1	54.9	15,495	63.4	36.6	15,495
Female spouse age groups	15–24	74.2	25.8	6685	86.1	13.9	6685
	25–34	62.1	37.9	20,184	77.2	22.8	20,184
	35+	46.7	53.3	24,928	66.1	33.9	24,928
Place of residence	Urban	54.3	45.7	12,465	68.2	31.8	12,465
	Rural	56.9	43.1	39,332	74.5	25.5	39,332
Education level of female spouse	No formal schooling	52.6	47.4	14,942	73.1	26.9	14,942
	Primary	54.8	45.2	7528	72.4	27.6	7528
	Secondary	58.0	42.0	24,066	73.2	26.8	24,066
	Higher	61.0	39.0	5261	72.7	27.3	5261
Education level of male spouse	No formal schooling	53.3	46.7	8507	74.5	25.5	8507
	Primary	55.2	44.8	7938	73.6	26.4	7938
	Secondary	56.8	43.2	28,115	72.8	27.2	28,115
	Higher	58.8	41.2	7237	71.4	28.6	7237
Religion	Hindu	58.0	42.0	39,655	73.2	26.8	39,655
	Muslim	51.4	48.6	5827	78.5	21.5	5827
	Others	50.2	49.8	6315	67.0	33.0	6315
Caste	Scheduled caste	58.7	41.3	9663	72.7	27.3	9663
	Scheduled tribe	53.6	46.4	10,373	72.6	27.4	10,373
	Other Backward Castes	58.3	41.7	19,924	73.8	26.2	19,924
	None of the above	53.8	46.2	9014	71.1	28.9	9014
Diet Index	Less diversified diet/unhealthy (unhealthy)	57.1	42.9	13,281	73.2	26.8	13,281
	Moderately diversified diet/moderate (Moderate)	56.2	43.8	25,635	72.6	27.4	25,635
	Diversified diet/healthy (Healthy)	55.6	44.4	12,881	73.7	26.3	12,881
Waist-to-Hip Ratio	Normal	63.0	37.0	11,452	79.5	20.5	11,452
	Female/Male spouses have high WHR	57.4	42.6	21,554	73.5	26.5	21,554
	Both spouses have high WHR	50.8	49.2	18,791	68.6	31.4	18,791
Male spouse occupation	Not working	54.8	45.2	1399	72.1	27.9	1399
	Agricultural	56.2	43.8	21,337	75.0	25.0	21,337
	Skilled and unskilled manual	57.9	42.1	14,096	73.9	26.1	14,096
	Others	55.0	45.0	14,848	69.5	30.5	14,848
Female spouse occupation	Not working	56.6	43.4	32,720	73.7	26.3	32,720
	Agricultural	56.5	43.5	10,782	74.0	26.0	10,782
	Skilled and unskilled manual	56.3	43.7	3267	69.0	31.0	3267
	Others	53.6	46.4	4974	68.8	31.2	4974
Spouse age gap in years	Up to 1	57.6	42.4	6660	75.5	24.5	6660
	2–3	56.2	43.8	15,209	75.7	24.3	15,209
	4–5	54.7	45.3	12,728	73.0	27.0	12,728
	6–7	56.0	44.0	7532	70.5	29.5	7532
	7+	57.7	42.3	9668	69.1	30.9	9668
Household wealth index	Poorest	59.1	40.9	10,708	78.2	21.8	10,708
	Poorer	56.4	43.6	11,542	75.7	24.3	11,542
	Middle	56.2	43.8	11,028	72.9	27.1	11,028
	Richer	55.6	44.4	10,011	70.2	29.8	10,011
	Richest	53.5	46.5	8508	66.3	33.7	8508
Indian regions	North	53.0	47.0	9866	71.7	28.3	9866
	Central	56.6	43.4	11,349	73.8	26.2	11,349
	East	61.2	38.8	8003	77.5	22.5	8003
	Northeast	49.6	50.4	7941	70.3	29.7	7941
	West	61.0	39.0	5985	77.8	22.2	5985
	South	59.0	41.0	5632	68.6	31.4	5632
**India**		**56.3**	**43.7**	**51,797**	**73.0**	**27.0**	**51,797**

Among couples in urban areas in India, higher hypertension prevalences were observed with 45.7 % for the female spouses and 31.8 % for the male spouses compared to their rural counterparts. Among religious groups, those categorized as "Others" had the highest prevalence of hypertension for both female (49.80 %) and male spouses (33.0 %). Couples where both partners had a high WHR showed a higher prevalence of hypertension, 49.2 % in females and 31.4 % in males, compared to couples where one of them had a high WHR. The prevalence of hypertension in males who were not working was higher compared to any other occupational categories, and among female spouses, those who were involved in skilled, unskilled manual work experienced higher prevalence. As the age gap between spouses increased, so did the hypertension prevalence among male spouses, but no such trend was observed in female spouses. There was a clear positive association between household wealth and the prevalence of hypertension for both female and male spouses. Overall, older age, lower education, unhealthy body composition, higher wealth, and certain regional and social factors were all associated with increased blood pressure in both male and female spouses (Table 1).

The most conspicuous finding was that, after controlling for background characteristics, both female and male spouses had higher odds of hypertension (OR = 1.19, p < 0.001, 95 % CI: 1.13–1.25, and OR = 1.30, p < 0.001, 95 % CI: 1.22–1.38, respectively) if one of the spouses had a high WHR compared to female and male spouses with normal WHR. However, the risk of hypertension increased further if both spouses had a high WHR. For instance, the odds of hypertension were 44 % higher in female spouses (OR = 1.44, p < 0.001, 95 % CI: 1.37–1.52) and 56 % higher in male spouses (OR = 1.56, p < 0.001, 95 % CI: 1.47–1.66), compared to female and male spouses with normal WHR (Table 2). As far as background characteristics are concerned, after controlling for other factors, age and wealth status showed a positive association with hypertension in female as well as male spouses. For instance, in case of female spouses, the risk increased by 44 % for the 25–34-year age group and 96 % for 35 years and older compared to female spouses in the 15–24-year age group. Similarly, for male spouses, the risk increased from 38 % for the 25–34-year age group to 150 % for the 45–54-year age group, compared to the 15–24-year age group of male spouses. In terms of wealth status, the risk for female spouses increased from 10 % for the poorer group to 23 % for the richest group compared to their poorest counterparts. Similarly, in male spouses, the risk increased from 7 % for the poorer group to 52 % for the richest group compared to their poorest counterparts. Another background variable that showed a consistent association with hypertension in both bivariate and multiple logistic regression is religion. For instance, the risk of hypertension increased for Muslim female spouses by 23 % compared to their Hindu counterparts, and for Muslim male spouses, it increased by 28 % compared to their Hindu counterparts.

**Table 2:** Adjusted odds ratios for the association between Waist-to-Hip ratio and other covariates with hypertension among married females and males in India, NFHS-5.

Variable		Hypertension in Female Spouse		Hypertension in Male Spouse	
		p-value	OR (95 % C.I.)	p-value	OR (95 % C.I.)
Male Spouse age groups	15–24				
	25–34	0.035	1.15 (1.01, 1.30)	0.000	1.38 (1.17, 1.64)
	35–44	0.000	1.57 (1.36, 1.81)	0.000	1.88 (1.56, 2.26)
	45–54	0.000	1.96 (1.67, 2.29)	0.000	2.50 (2.05, 3.05)
Female spouse age groups	15–24				
	25–34	0.000	1.44 (1.33, 1.56)	0.000	1.36 (1.23, 1.50)
	35+	0.000	1.96 (1.77, 2.17)	0.000	1.71 (1.51, 1.93)
Place of residence	Urban				
	Rural	0.106	0.96 (0.91, 1.01)	0.004	0.92 (0.87, 0.97)
Educational level of female spouse	No formal schooling				
	Primary	0.818	0.99 (0.93, 1.06)	0.350	1.03 (0.96, 1.11)
	Secondary	0.166	0.96 (0.91, 1.02)	0.775	0.99 (0.93, 1.06)
	Higher	0.000	0.84 (0.76, 0.92)	0.050	0.90 (0.82, 1.00)
Educational level of male spouse	No formal schooling				
	Primary	0.614	0.98 (0.92, 1.05)	0.009	1.11 (1.03, 1.2)
	Secondary	0.249	0.96 (0.91, 1.03)	0.001	1.13 (1.06, 1.21)
	Higher	0.025	0.91 (0.83, 0.99)	0.048	1.10 (1.00, 1.22)
Religion	Hindu				
	Muslim	0.000	1.23 (1.15, 1.33)	0.000	0.72 (0.66, 0.78)
	Others	0.099	1.06 (0.99, 1.14)	0.000	1.20 (1.11, 1.30)
Caste	Scheduled caste				
	Scheduled tribe	0.000	1.16 (1.08, 1.24)	0.017	1.10 (1.02, 1.18)
	Other Backward Castes	0.449	0.98 (0.93, 1.03)	0.002	0.91 (0.85, 0.97)
	None of above	0.041	1.07 (1.00, 1.14)	0.502	0.98 (0.91, 1.05)
Waist-to-Hip Ratio (WHR)	Normal WHR				
	Female/Male spouse have high WHR	0.000	1.19 (1.13, 1.25)	0.000	1.30 (1.22, 1.38)
	Both spouses have high WHR	0.000	1.44 (1.37, 1.52)	0.000	1.56 (1.47, 1.66)
Diet Index	Less diversified diet/unhealthy (unhealthy)				
	Moderately diversified diet/moderate (Moderate)	0.235	1.03 (0.98, 1.09)	0.008	1.08 (1.02, 1.14)
	Diversified diet/healthy (Healthy)	0.947	1.00 (0.94, 1.06)	0.048	1.07 (1.00, 1.15)
Male spouse occupation	Not working				
	Agricultural	0.374	0.95 (0.84, 1.07)	0.196	0.92 (0.80, 1.05)
	Skilled and unskilled manual	0.269	0.93 (0.83, 1.05)	0.627	0.97 (0.84, 1.11)
	Others	0.577	0.97 (0.86, 1.09)	0.374	1.06 (0.93, 1.22)
Female spouse occupation	Not working				
	Agricultural	0.027	0.94 (0.89, 0.99)	0.580	0.98 (0.93, 1.04)
	Skilled and unskilled manual	0.278	0.96 (0.88, 1.04)	0.000	1.17 (1.07, 1.28)
	Others	0.552	1.02 (0.95, 1.09)	0.751	1.01 (0.94, 1.09)
Spouse age gap in years	Up to 1				
	2–3	0.969	1.00 (0.94, 1.06)	0.051	0.93 (0.86, 1.00)
	4–5	0.445	0.97 (0.91, 1.04)	0.717	0.99 (0.91, 1.06)
	6–7	0.056	0.93 (0.86, 1.00)	0.097	1.08 (0.99, 1.17)
	7+	0.000	0.85 (0.78, 0.91)	0.035	1.10 (1.01, 1.20)
Household wealth index	Poorest				
	Poorer	0.001	1.10 (1.04, 1.17)	0.045	1.07 (1.00, 1.15)
	Middle	0.000	1.14 (1.07, 1.22)	0.000	1.21 (1.13, 1.31)
	Richer	0.000	1.17 (1.08, 1.26)	0.000	1.33 (1.22, 1.44)
	Richest	0.000	1.23 (1.13, 1.34)	0.000	1.52 (1.38, 1.68)
Indian regions	North				
	Central	0.727	0.99 (0.93, 1.05)	0.001	1.12 (1.04, 1.20)
	East	0.000	0.77 (0.72, 0.83)	0.000	0.83 (0.76, 0.90)
	Northeast	0.000	1.21 (1.12, 1.32)	0.192	1.06 (0.97, 1.16)
	West	0.000	0.81 (0.75, 0.87)	0.000	0.81 (0.74, 0.88)
	South	0.000	0.83 (0.77, 0.90)	0.013	1.11 (1.02, 1.21)

Table 3 is included to shed light on important contributing factors to high WHR, since it emerged to be an important risk factor for hypertension in male and female married spouses. It revealed that as age increased in both male and female spouses, the WHR also increased among couples. For instance, if the male spouses belonged to the 15–24-year age group, the corresponding high WHR prevalence was 22.8 % and it increased to 40.8 % in the 45–54-year age group. Similarly, if the female spouses belonged to the 15–24 age group, the corresponding high WHR prevalence was 28.3 % and it increased to 40.0 % in the 35+ years age group. According to place of residence, 42.3 % in rural areas and 39.6 % in urban areas had one of the spouses with high WHR prevalence. However, 42.5 % of urban couples and 34,3 % rural couples had both spouses with high WHR. The high WHR prevalence increased for couples with education, from 33.9 % to 42,1 % for female spouses with no education to those with higher secondary education. Similarly, the high WHR prevalence increased for couples with education, from 32.7 % to 40.4 % for male spouses with no education to those with higher secondary education. Muslims showed the highest couple-level high WHR prevalence (44.2 %) compared to any other religion, while Scheduled Tribes had the highest prevalence compared to any other caste in one of the spouses individually (43.0 %), but the lowest among couples (29.7 %). There was a positive association between the household wealth index and couples’ high WHR prevalence, increasing from 28.7 % in the poorest wealth quintile to 45.9 % in the richest wealth quintile.

**Table 3:** Prevalence of Waist-to-hip ratio by background characteristics among married couples in India, NFHS-5.

Variable		Waist-to-Hip Ratio			
		Normal	Female/Male spouse have high WHR	Both spouses have high WHR	Total
Male Spouse age groups	15–24	35.6	41.6	22.8	2137
	25–34	26.0	41.9	32.1	14,890
	35–44	20.7	41.9	37.4	19,275
	45–54	18.2	41.0	40.8	15,495
Female spouse age groups	15–24	30.2	41.5	28.3	6685
	25–34	23.3	42.3	34.3	20,184
	35+	18.9	41.1	40.0	24,928
Place of residence	Urban	18.0	39.6	42.5	12,465
	Rural	23.4	42.3	34.3	39,332
Educational level of female spouse	No formal schooling	23.5	42.6	33.9	14,942
	Primary	23.1	42.9	34.1	7528
	Secondary	21.9	41.0	37.2	24,066
	Higher	17.9	40.0	42.1	5261
Educational level of male spouse	No formal schooling	24.9	42.4	32.7	8507
	Primary	24.1	43.1	32.9	7938
	Secondary	21.7	41.1	37.3	28,115
	Higher	18.4	41.2	40.4	7237
Religion	Hindu	23.0	42.2	34.8	39,655
	Muslim	17.5	38.4	44.2	5827
	Others	21.0	40.9	38.1	6315
Caste	Scheduled caste	21.2	40.3	38.5	9663
	Scheduled tribe	27.3	43.0	29.7	10,373
	Other Backward Castes	22.6	42.8	34.6	19,924
	None of above	17.4	39.7	42.9	9014
Diet Index	Less diversified diet/unhealthy (unhealthy)	22.6	41.9	35.5	13,281
	Moderately diversified diet/moderate (Moderate)	21.3	41.3	37.4	25,635
	Diversified diet/healthy (Healthy)	23.2	41.9	34.9	12,881
Male spouse occupation	Not working	22.3	39.0	38.7	1399
	Agricultural	25.5	42.6	31.8	21,337
	Skilled and unskilled manual	21.4	42.0	36.6	14,096
	Others	17.9	40.0	42.1	14,848
Female spouse occupation	Not working	20.7	41.1	38.3	32,720
	Agricultural	28.3	43.4	28.4	10,782
	Skilled and unskilled manual	21.5	41.5	36.9	3267
	Others	18.7	41.4	39.9	4974
Spouse age gap in years	Up to 1	22.8	41.3	35.9	6660
	02–03	23.4	41.3	35.3	15,209
	04–05	22.4	41.3	36.3	12,728
	06–7	20.4	43.1	36.6	7532
	7+	20.7	41.5	37.8	9668
Household wealth index	Poorest	27.7	43.6	28.7	10,708
	Poorer	24.9	42.1	33.0	11,542
	Middle	22.2	41.4	36.4	11,028
	Richer	18.8	41.3	39.9	10,011
	Richest	15.1	39.0	45.9	8508
Indian regions	North	14.8	38.6	46.7	9866
	Central	24.9	43.3	31.8	11,349
	East	17.9	39.9	42.2	8003
	Northeast	22.5	41.8	35.7	7941
	West	33.4	42.5	24.2	5985
	South	21.2	43.8	35.0	5632

## 4 Discussion

This cross-sectional study, based on a total sample of 51,797 couples from recently conducted nationally representative sample data, was analysed using bivariate and multivariate techniques. The most important finding was that high WHR prevalence is strongly associated with higher odds of hypertension in both spouses. After controlling for confounding background characteristics, both female and male spouses had higher odds of hypertension when one of the spouses had a high WHR prevalence compared to those with a normal WHR. The risk increased to 44 % for the female spouses and 56 % for the male spouses compared to the couples with normal WHR when both spouses had high WHR prevalence. This is an unequivocal support for the primary objective of the paper that high WHR prevalence would be a strong risk factor among Indian couples. These findings align with studies conducted by Deng et.al (2018) in China and Lee et.al (2015) in Korea, which have shown that being overweight, as indicated by a high WHR, is a key predictor of hypertension [[22], [23]]. A study conducted in 2014 by Bajaj et.al compared relative waist circumference between Asian Indians and US adults, and indicated that there is clearly more variation in the waist circumference of Asian Indians than in the US adults for any given body size. It was further mentioned that the waist-to-hip ratio was threefold higher among Indian men and women compared to that of the US men and women. This observation suggests that Asian Indians may have a heightened propensity for accumulating central adiposity, for any body size, compared to other populations [24].

The risk of hypertension increased with household wealth; the richest group showed the highest risk of 23 % for female spouses and 52 % in the case of male spouses, compared to the couples that belonged to the poorest group. According to a recent study by Chaudhary and Sharma (2023), increasing affluence leads to shared sedentary behaviors and unhealthy diets within households, contributing to high WHR, and this in turn increases the risk of hypertension among couples [25]. This supports the “nutrition transition” theory explanation advanced by Popkin (1999). This theory also suggests a possible positive association between wealth and the prevalence of high WHR among Indian couples, which can lead to increased hypertension risk [26].

We have considered married couples and their ages for the study. This is partly because a study conducted in China by Liu et.al (2021) has shown that marriage contributes to a higher risk for being overweight and it is likely to increase the risk for hypertension [5]. Other researchers, such as Jacobson (2007), also found that couples who resemble in body mass and in turn have a higher risk for hypertension [27]. We have observed in our study that age is positively associated with WHR as well as with hypertension. The study by Gordon-Larsen (2009) also found that couples who cohabitate for long periods of time are more likely to show concordance in behaviors associated with being overweight because of low levels of physical activity and a generally sedentary lifestyle, likely to lead to a higher risk of hypertension [28].

One of the variables that showed a strong positive association with the prevalence of hypertension among Indian couples, both in bivariate and multivariate analyses, was Muslim religious background. In the data set, we have also seen that Muslim dietary cultures centred around high intake of animal foods—rich in red meat and fats— which may have been directly linked to increased WHR among Muslim couples in India. According to Sahay et.al (2023), community and religious festivals such as Eid and Muharram, among Indian Muslims, often involve calorie-dense meals [29].

It is necessary to highlight a few strengths of our study. First, the multistage stratified cluster random sampling method used in NFHS-5 ensured a high degree of representativeness. The questionnaire was based on the experience of Demographic Health Surveys of many countries, and the training of the interviewers was carefully planned based on the past four NFHS surveys to obtain data of high quality. The survey’s response rate was also high. Secondly, the study of the spousal double burden of WHR stratified each variable at the couple level. We analysed the differences in WHR, association, and consistency values of each subgroup. Third, our study also analysed the differences in the risks between husbands and wives due to various socioeconomic and anthropometric factors.

This study has certain limitations. First, the cross-sectional design precludes causal inference. Second, the reliance on self-reported data for occupation and education may introduce reporting bias or misclassification, especially in settings with informal or overlapping employment roles. Additionally, data on the duration of marriage—a key factor influencing the degree of spousal concordance in health behaviors and outcomes—was not available in the NFHS-5 dataset. Future research should consider incorporating marriage duration and conducting longitudinal or mixed-method studies to explore how shared environmental exposures, chronic stress, co-regulated dietary habits, and physical activity patterns contribute to the concordance in central obesity and hypertension among couples.

To conclude, marriage is generally believed to promote health; it can also lead to poorer health and overweight, leading to hypertension, especially in certain groups, such as the rich, Muslims, and couples in the north-east regions. Therefore, it is necessary to promote healthy dietary practices and physical activity in the high-risk groups. Government health services should implement health education activities that encourage people to change their behavior to reduce high WHR, thereby reducing the risk of hypertension associated with high WHR, and create a healthy family living environment for the next generation. As recommended by Ross et.al (2020) and Consultation (2008), primary healthcare providers routinely assess WHR alongside BMI and blood pressure, as per the International Atherosclerosis Society (IAS) and the International Collaboration of Cardiovascular Research (ICCR) Consensus Guidelines, which emphasize the clinical utility of WHR in assessing visceral obesity and hypertension risks in South Asian populations [[30], [31]]. Furthermore, the development of couple-focused lifestyle intervention programs—addressing physical activity, dietary modification, and behavioral counselling—may offer synergistic benefits, improve adherence, and reduce household-level hypertension risk. These strategies could enhance the real-world applicability of our findings and support more inclusive family-based prevention models.

## Data availability statement

The datasets analysed during the current study are available in the Demographic and Health Survey repository (https://dhsprogram.com/data/).

## Ethical statement

The study performed a secondary analysis, and there was no identifiable information about the respondents. Hence, ethical approval was not required.

## Funding statement

This work did not receive any funding.

## CRediT authorship contribution statement

**Rajeshwari A. Biradar:** Writing – review & editing, Writing – original draft, Visualization, Validation, Software, Project administration, Methodology, Formal analysis, Data curation, Conceptualization. **Jang Bahadur Prasad:** Writing – review & editing, Validation, Methodology, Formal analysis, Conceptualization. **Shiva S Halli:** Writing – review & editing, Funding acquisition, Conceptualization.

## Declaration of competing interest

The authors declare the following financial interests/personal relationships which may be considered as potential competing interests: Shiva S Halli reports was provided by University of Manitoba, Winnipeg. Shiva S Halli reports a relationship with University of Manitoba, Winnipeg that includes:. Shiva S Halli has patent issued to NA. NA If there are other authors, they declare that they have no known competing financial interests or personal relationships that could have appeared to influence the work reported in this paper.
